# A consensus based template for reporting of pre-hospital major incident medical management

**DOI:** 10.1186/1757-7241-22-5

**Published:** 2014-01-30

**Authors:** Sabina Fattah, Marius Rehn, David Lockey, Julian Thompson, Hans Morten Lossius, Torben Wisborg

**Affiliations:** 1 Department of Research and Development, Norwegian Air Ambulance Foundation, Drøbak, Norway; 2 Anaesthesia and Critical Care Research Group, Faculty of Health Sciences, University of Tromsø, Tromsø, Norway; 3 Field of Pre-hospital Critical Care, Network of Medical Sciences, University of Stavanger, Stavanger, Norway; 4 Department of Anesthesiology and Intensive Care, Akershus University Hospital, Lørenskog, Norway; 5 School of Clinical Sciences, University of Bristol, Bristol, UK; 6 London’s Air Ambulance, The Helipad, Royal London Hospital, Whitechapel, London, UK; 7 Department of Anaesthesiology and Intensive Care, Hammerfest Hospital, Finnmark Health Trust, Hammerfest, Norway; 8 Norwegian Trauma Competency Service, Oslo University Hospital, Oslo, Norway

**Keywords:** Major incident, Disaster, Emergency medicine, Reporting, Medical management

## Abstract

**Background:**

Structured reporting of major incidents has been advocated to improve the care provided at future incidents. A systematic review identified ten existing templates for reporting major incident medical management, but these templates are not in widespread use. We aimed to address this challenge by designing an open access template for uniform reporting of data from pre-hospital major incident medical management that will be tested for feasibility.

**Methods:**

An expert group of thirteen European major incident practitioners, planners or academics participated in a four stage modified nominal group technique consensus process to design a novel reporting template. Initially, each expert proposed 30 variables. Secondly, these proposals were combined and each expert prioritized 45 variables from the total of 270. Thirdly, the expert group met in Norway to develop the template. Lastly, revisions to the final template were agreed via e-mail.

**Results:**

The consensus process resulted in a template consisting of 48 variables divided into six categories; pre-incident data, Emergency Medical Service (EMS) background, incident characteristics, EMS response, patient characteristics and key lessons.

**Conclusions:**

The expert group reached consensus on a set of key variables to report the medical management of pre-hospital major incidents and developed a novel reporting template. The template will be freely available for downloading and reporting on http://www.majorincidentreporting.org. This is the first global open access database for pre-hospital major incident reporting. The use of a uniform dataset will allow comparative analysis and has potential to identify areas of improvement for future responses.

## Background

Major incidents such as natural disasters, complex road traffic accidents, terrorism attacks and violence in general, are global problems. Over the decade 2001–2010, an average of more than 700 natural and technological emergencies occurred globally every year, affecting approximately 270 million people and causing over 130 000 deaths annually [1]. In 2011 natural disasters alone cost more than 30 000 lives and caused some 245 million victims worldwide [2]. Road traffic injury (RTI) is a global public health problem causing some 1,2 million deaths yearly and another 20–50 million people sustain non-fatal injuries. RTI rates are twice as high in low-and middle- income countries compared to high-income countries [3]. Further, terrorism caused over 86 000 injured and some 25 000 fatalities in the period from 1968 until 2004 [4]. Conflict-related emergencies are yet another challenge affecting over 1.5 billion people or one quarter of the world’s population who live in countries affected by violent conflict [5].

In the last sixty years disaster medicine has been recognised as a distinct scientific discipline [6]. However the medical reporting of major incidents has been inconsistent leading to several calls for more structured reporting [7-11]. A systematic review to identify templates for reporting major incident medical management revealed that 10 such templates exist globally [12]. The templates were heterogeneous and their implementation has been limited. Further, no feasibility testing has been performed.

Current literature identifies challenges in major incident medical management such as communication [13,14], coordination [15], triage [16,17] and distribution of patients [18]. We aim to address the challenges by designing a template that is feasible and freely accessible to allow rapid dissemination of information for practical and comparative analysis. Based on a modified nominal group technique, we conducted a consensus process to identify data variables that should be incorporated into such a template.

## Methods

### Definition

Major incident was defined as ‘an incident that requires the mobilization of extraordinary EMS resources and is identified as a major incident in that system’.

### The experts

European experts who had published previous major incident reporting templates were identified through a systematic literature review [12] and were invited to participate. Six authors were identified and four were able to take part in the consensus process. The organizers were each asked to nominate two experts with experience as a major incident practitioner, planner or academic. Nine nominated experts were able to participate. In total 13 experts from 10 European countries participated.

### The modified nominal group technique

The four-stage consensus process was based on the Nominal Group Technique [19] modified according to the experience gained by researchers in the Norwegian Air Ambulance Foundation in undertaking recent consensus processes [20-24]. The process consisted of three written stages where experts worked individually and one collective meeting with verbal negotiations. The process began in December 2012 and final modifications were made in October 2013.

### Stage 1

The experts were each asked to suggest 30 data variables that they believed to be of greatest value concerning pre-hospital major incident medical management reporting.

### Stage 2

One month later, the experts were asked to choose the 45 most important variables from all suggested variables in stage 1. The reason for choosing 45 variables was to prevent the experts from only choosing their 30 suggested variables from stage 1. During this stage experts were also allowed to combine variables considered to have the same core meaning. The 45 variables suggested by each expert were given a point value: a ranking of first place gave 45 points, second place 44 points and so on until the priority on 45th place received 1 point. In addition each suggested variable received 2 points for every time it was nominated in an expert’s top 45 lists.

A month later a list containing the variables that scored more than 100 points together with their comments was sent to the experts. This step allowed the experts to perform a second examination of relevant scientific material prior to the consensus meeting.

### Stage 3

Two weeks later the expert group attended a 2-day meeting in Torpomoen, Norway. The highest ranked variables were discussed and a draft of the final template agreed upon. Variables and definitions were collated with existing Utstein templates for reporting from trauma care and major incidents [22,25].

### Stage 4

The organisers edited this draft into a consistent structure and circulated it to the experts for final revision two weeks after the consensus. The group undertook revisions in August 2013. Experts with experience in testing questionnaires for Statistics Norway reviewed the template and provided suggestions for improvement from a user point-of-view. Most of these suggestions were incorporated into the template before it was distributed to the consensus group for final approval in October 2013.

## Results

Stage 1 resulted in 339 suggested data variables that were categorized without modifying the experts’ suggestions. Only identical or very similar variables were merged, resulting in a total list of 270 variables. Stage 2 resulted in a list of 41 variables that scored more than 100 points. These were discussed at the consensus meeting and resulted in a template consisting of 48 variables each allocated into one of six categories to create a structure for the final template (Additional file 1: Printer friendly version of template).

### Pre-incident data

This section gives the reader a brief overview of the geographical setting and infrastructure in the affected area before the incident occurred. It will ask for information such as the population and population density, pre-existing infrastructure stating accessibility in the area (by road, train, boat, foot) and the telecommunications network. It will also allow the author to provide information on specific local issues, such as civil unrest or political situation.

### Emergency medical system (EMS) background

These variables aim to describe pre-incident EMS characteristics in the affected area before the incident, and will allow the reader to evaluate its relevance to their own EMS system. The data includes information on the EMS, response activation, staffing of ambulance services, availability of resources, triage and major incident training. Variables describing staffing of ambulance services were modified from a previous template [21] (Additional file 1: Questions 1-11).

### Incident characteristics

This section consists of eight variables pertaining to incident background, access, evacuation of patient, infrastructure damage, sites with separate EMS infrastructures and hazards. These variables will allow users of the database to stratify incidents by type (e.g. earthquake, nuclear accident) and enables comparative analysis of incidents within the same category (Additional file 1: Questions 12-19).

### EMS response data

A previously published template [25] influenced variables concerning EMS response: initial actions by first medical team, medical coordination, medical communications and medical command structure. Variables concerning timings and hospitals receiving patients are similar to another existing template [26]. Other data in this section are: personnel, transport and material resources on scene and data on patient surge. Many of these variables will be considered quality indicators that will not only describe the response, but also allow researchers to compare medical response, and identify strengths and weaknesses (Additional file 1: Questions 20-32).

### Patient characteristics

The variables include population at risk from the incident and actual casualties, gender, number of dead and patient distribution. The patient distribution variables include both EMS response data (surge data) and patient characteristics (triage data). Paediatric patients were subcategorized according to existing age categories [27]. The aim of these variables will be to identify factors that may affect patient mortality and morbidity (Additional file 1: Questions 33-46).

### Key lessons

This section allows the report author to communicate the key successes and problems in the major incident medical response and give the readers an overview of main lessons. For research purposes this section together with the first category will provide data for qualitative analysis (Additional file 1: Questions 47-48).

### Online reporting

**Figure 1:**
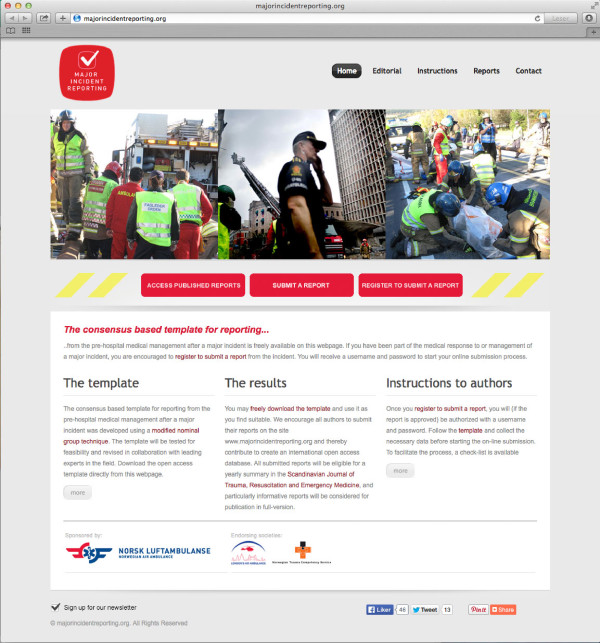
**Front page of http://www.majorincidentreporting.org.** The first global open access webpage for reporting from major incidents and accessing existing reports.

Following the consensus process, a webpage allowing online reporting using the template has been developed. The template can be accessed, freely downloaded and reports submitted free of article processing charge on: http://www.majorincidentreporting.org (Figure 1). The editorial process for submitted reports will be described on the webpage.

## Discussion

Through this consensus process, a group of European major incident experts have developed a template for the global reporting from pre-hospital major incident medical management. The authors of several existing templates contributed to this process aiming to create a practical and accessible template focused on the pre-hospital phase of major incident response. The template consisting of 48 variables in 6 categories can be completed and freely accessed online. An aim is that the template be widely implemented and accessible. It will be feasibility tested and revised in collaboration with experts working in this field.

### The data variables and outcome

Informed scientific evaluation of the impact of pre-hospital interventions on patient outcomes is vital [28]. Measures of outcome used in previous studies of daily EMS have been analysed according to the six Ds: death, disease, discomfort, disability, dissatisfaction and debt (cost). Death and disease were the most common outcomes evaluated and the other 4 Ds were infrequently measured [29]. Little is published regarding the validity, reliability and responsiveness of instruments for measuring outcome following major trauma [30]. In the template 30-day mortality is included, however different definitions influence how performance outcome is evaluated [31]. The template also includes data on proxy outcomes such as triage, surge and safety on site that reflect the immediate major incident medical management without being influenced by other phases such as the hospital phase and rehabilitation.

### Implementation of the template

The template will be implemented using an online database http://www.majorincidentreporting.org.

Using this template and contributing to creating an open access global database for reporting major incidents is an act of solidarity towards improving the outcome of disasters. The template is intentionally focused upon the variables that the expert group believes are likely to be of most importance to future incidents. The template content and availability of a database for reporting aims to reduce the threshold for reporting and increase global capture of critical information. In addition to the humanitarian aspect in the development of a global major incident database and dissemination of key lessons, we aim to maximise contribution by waiving the fee for report submission. The reporting of experiences through the website should not prevent individual publications in other journals.

### Ethical considerations

The template has been created to avoid compromising patient confidentiality, therefore no identifiable patient data will be reported to the database nor will there be the facility to upload images. Pre-approval from ethics committees to access data necessary for filling in the template would be preferable to allow reporting to take place quickly after an incident and prevent time delay in disseminating relevant knowledge to others. However, it is uncertain how practical it will be to obtain such ethics approvals. The greatest impact of major incidents in the form of natural disasters are in low-and middle-income countries [2], the same applies for road traffic accidents [3]. Due to these facts it is morally and scientifically important that a template be available and relevant for reporting and analysis also in these areas. Whether this is the case for this template will be sought answered in a feasibility study.

### Strengths and limitations

Using a nominal group technique consensus process may be a limitation with regards to selection of participants and wording of the question influencing the outcome [19]. The composition of experts ensures a valid mix of practical and theoretical approach to major incident management. Stages 1 and 2 ensured that each expert opinion was equally weighted in the nomination of variables. Disaster terminology is yet another challenge [32] and various definitions exist [33-35]. Our definition of a major incident aligns with previous definitions [36], and aims to be easily comprehensible.

Accurate data collection in extreme circumstances may be challenging and may be reflected in erroneous data collection. Moreover there may be difficulties in gaining complete data capture following incidents particularly when security, military and political sensitivities are involved or infrastructure damage is such that no data collection occurs. The database will not provide the basis for calculating denominators and nominators for use in major incident epidemiology, for this purpose, mandatory national registries are necessary [37]. These issues as well as feasibility regarding the type and amount of data to be reported, and whether including only European experts in this process was a limitation will be addressed in feasibility studies.

## Conclusions

Consensus was achieved amongst experts on key data variables for reporting the pre-hospital major incident medical management. The template is the basis for the first global open access database for major incidents and is available for downloading and reporting on http://www.majorincidentreporting.org. The use of a uniform dataset after each major incident will allow for comparative analysis to take place and aims to identify improvements for future medical response. We invite those directly involved in the response to or management of a previous or future major incident to freely use the template and publish reports open access.

## Competing interests

The authors declare no competing interests.

## Authors’ contributions

SF, MR, DL, JT, HML and TW all participated in designing the study, analysis of data and organization of the process. All authors and collaborators approved the final version of the manuscript.

## Supplementary Material

Additional file 1 Pdf printer friendly version of template for reporting pre-hospital major incident medical management. Click here for file
