# Erratum: a consensus based template for reporting of pre-hospital major incident medical management

**DOI:** 10.1186/s13049-014-0042-6

**Published:** 2014-09-06

**Authors:** Sabina Fattah, Marius Rehn, David Lockey, Julian Thompson, Hans Morten Lossius, Torben Wisborg, The Major Incidence Reporting Collaborators

**Affiliations:** Department of Research and Development, Norwegian Air Ambulance Foundation, Drøbak, Norway; Anaesthesia and Critical Care Research Group, Faculty of Health Sciences, University of Tromsø, Tromsø, Norway; Field of Pre-hospital Critical Care, Network of Medical Sciences, University of Stavanger, Stavanger, Norway; Department of Anesthesiology and Intensive Care, Akershus University Hospital, Lørenskog, Norway; School of Clinical Sciences, University of Bristol, Bristol, UK; London’s Air Ambulance, The Helipad, Royal London Hospital, Whitechapel London, UK; Department of Anaesthesiology and Intensive Care, Hammerfest Hospital, Finnmark Health Trust, Hammerfest, Norway; Norwegian Trauma Competency Service, Oslo University Hospital, Oslo, Norway

## Erratum

After the publication of our article “A consensus based template for reporting of pre-hospital major incident medical management” Scand J Trauma Resusc Emerg Med 2013, 22:5, we noticed that The Major Incident Reporting Collaborators were not included as authors. They have now been added at the end of the author list. The group consists of Gareth Davies, Michel Debacker, Erik Frischknecht Christensen, Juhana Hallikainen, Troels Martin Hansen, Jorine Juffermans, Per Kulling, Vidar Magnusson, Jannicke Mellin-Olsen, Kai Milke, Anders Ruter, Stephen JM Sollid, Wolfgang Voelckel.

