# Supplementary material for: A consensus based template for reporting of pre-hospital major incident medical management
Source: Scand J Trauma Resusc Emerg Med. 2014 Jan 30;22:5. doi: 10.1186/1757-7241-22-5 (PMC3922248; doi:10.1186/1757-7241-22-5)
Supplement: Additional file 1 — Pdf printer friendly version of template for reporting pre-hospital major incident medical management. [file 1757-7241-22-5-S1.PDF]

MAJOR INCIDENT REPORTING

NORSK LUFTAMBULANSE
NORWEGIAN AIR AMBULANCE

LONDON'S AIR AMBULANCE

# Template for reporting pre-hospital medical major incident management

○ Indicates that only one option can be ticked.
□ Indicates that several options can be ticked.

## Pre-incident data

Free text. Maximum number of words: 500.
Please provide following minimum information: in which country/ies did the major incident occur. The population and population density (number of people living per unit of an area) in the affected area. Information on pre-existing infrastructure stating accessibility in the area (by road, train, boat, foot) and the telecommunications network. You should also describe any special conditions in the country and area.

# EMS background

| | |
|---|---|
| 1 | **Was an EMS coordinating centre (the centre responsible for dispatching and coordinating EMS units on-scene) available in the affected country/ies before the incident?**<br>○ Yes<br>○ No |
| 2 | **Does a dialling number to Emergency Services exist?**<br>○ Yes<br>○ No<br>*If yes: a single and unique dialling number to EMS or one common dialling number for all Emergency Services (fire, police and EMS)?*<br>*If no: please specify how EMS is alerted.* |
| 3 | **Can a major incident be declared directly by the person receiving an alert at the EMS coordinating centre?**<br>○ Yes<br>○ No<br>○ Unknown |
| 4 | **What is the background of staff in the every-day/normal staffing of EMS services? Please tick for all options that apply.**<br>□ Basic Life Support by non-EMS professional<br>□ Basic Life Support by EMS professionals, non-physician<br>□ Advanced Life Support by EMS professional, non-physician<br>□ Advanced Life Support on-scene by physician<br>□ Other/unknown<br>*If other please specify.* |
| 5 | **What other resources are routinely available to assist the EMS service in a normal setting? Please tick for all options that apply.**<br>□ Fire brigade<br>□ Police<br>□ Voluntary organizations.<br>□ Coast guard<br>□ Military<br>□ Civil protection<br>□ Other/unknown<br>*If other please specify.*<br>*If voluntary organizations are available please specify which and if these require authorisation from police or other authorities to participate in the response phase.* |

| | |
|---|---|
| 6 | **What other resources can be mobilized in a major incident? Please tick for all options that apply.**<br>☐ Fire brigade<br>☐ Police<br>☐ Voluntary organizations<br>☐ Coast guard<br>☐ Military<br>☐ Civil protection<br>☐ Other/unknown<br>*If other please specify.*<br>*If voluntary organizations are available please specify which and if these require authorisation from police or other authorities to participate in the response phase.* |
| 7 | **How many and what type of hospitals exist within the EMS catchment system that was affected by the major incident? Please tick for all options that apply.**<br>☐ Regional hospital with trauma specialty<br>☐ Regional hospital without trauma specialty<br>☐ Local hospital without trauma specialty<br>☐ Other types of hospitals. Please specify<br>*Please state whether the numbers are estimated or exact.* |
| 8 | **Is a pre-hospital on-scene triage system in use daily on a national level?**<br>○ Yes<br>○ No<br>○ Unknown<br>*If yes please specify which triage system/s.*<br>**Is a pre-hospital on-scene triage system in use daily on regional levels?**<br>○ Yes<br>○ Yes, but different triage systems exist in different regions<br>○ No<br>○ Unknown<br>*If yes please specify which triage system/s.* |
| 9 | **Is a pre-hospital on-scene triage system for major incidents in use on a national level?**<br>○ Yes<br>○ No<br>○ Unknown<br>*If yes please specify which triage system/s.*<br>**Is a pre-hospital on-scene triage system for major incidents in use on regional levels?**<br>○ Yes<br>○ Yes, but different triage systems exist in different regions<br>○ No<br>○ Unknown<br>*If yes please specify which triage system/s.* |
| 10 | **Does the pre-hospital on-scene triage system for major incidents include direct tagging/labelling of patients?**<br>○ Yes<br>○ No<br>○ Unknown<br>○ N/A |
| 11 | **For those employees within the pre-hospital EMS system who are intended to work on-scene: is major incident training mandatory?**<br>○ Yes<br>○ No<br>○ Unknown |

# Incident characteristics

| | |
|---|---|
| 12 | **What was the mechanism/external factor that caused the incident? Please tick for all options that apply.**<br>☐ Transport accident<br>☐ Extreme weather<br>☐ Seismic incident<br>☐ Fire<br>☐ Mass gathering<br>☐ Explosive<br>☐ Industrial accident<br>☐ Nuclear or radiological incident<br>☐ Biological incident<br>☐ Chemical incident<br>☐ Other. Please specify<br>*If extreme weather please choose one of the options below:*<br>☐ Avalanche<br>☐ Flooding<br>☐ Thunderstorm<br>☐ Hurricane<br>☐ Extreme heat<br>☐ Extreme cold<br>☐ Other. Please specify<br><br>**Is this incident coupled to another incident?**<br>○ Yes<br>○ No<br>*If yes, which incident?* |

| | |
|---|---|
| 13 | **What was the location of the incident scene? Please tick for all options that apply.**<br>☐ Urban area<br>☐ Rural/ countryside area<br>☐ Offshore/ maritime (ocean, river, lake)<br>☐ Mountain<br>☐ Road<br>☐ Airport<br>☐ Educational facility<br>☐ Public facility<br>☐ Health care facility<br>☐ Building<br>☐ Mass gathering<br>☐ Other/unknown<br>*If other please specify.* |
| 14 | **What was the EMS' mode of access to treat patients at incident scene? Please tick for all options that apply.**<br>☐ Wheeled vehicles<br>☐ Rail<br>☐ Air<br>☐ Boat<br>☐ Foot<br>☐ Other. Please specify<br>*For each ticked alternative please specify if there was a delay in accessing the patient in order to start evaluation/treatment and why this occured.*<br>*Reasons for delay could include reasons such as: security issues, congested roads due to traffic, weather conditions.* |
| 15 | **What was the EMS' mode of evacuating patients from the incident scene? Please tick for all options that apply.**<br>☐ Wheeled vehicles<br>☐ Rail<br>☐ Air<br>☐ Boat<br>☐ Foot<br>☐ Other. Please specify<br>*For each ticked alternative please specify if there was a delay in the evacuation of the patient/s and why this occured.*<br>*Reasons for delay could include reasons such as: entrapment, lack of transport capacity, weather conditions etc.* |
| 16 | **Was there damage to infrastructure that affected EMS response? Please tick for all options that apply.**<br>☐ Power<br>☐ Telecommunication<br>☐ Other modes of communication<br>☐ Road<br>☐ Rail<br>☐ Damage to the EMS or health structure<br>☐ Other damage. Please specify<br>*For each ticked alternative please state what the damage was and how it affected the EMS response.* |
| 17 | **How many sites required separate EMS infrastructure (such as on-scene leadership and casualty clearing stations) in the response phase?**<br>Please state whether the number is estimated or exact. |
| 18 | **Which hazards existed for rescuers on scene? Please tick for all options that apply.**<br>☐ On going violence or risk of further violence<br>☐ Fire<br>☐ Collapsing building/s<br>☐ Climate<br>☐ Lack of electricity<br>☐ Lack of water/food<br>☐ Other. Please specify<br>*For each ticked alternative please specify what the hazard was and how it affected the rescuers on-scene.* |
| 19 | **Which hazards existed for patients on scene? Please tick for all options that apply.**<br>☐ On going violence or risk of further violence<br>☐ Fire<br>☐ Collapsing building/s<br>☐ Climate<br>☐ Lack of electricity<br>☐ Lack of water/food<br>☐ Other. Please specify<br>*Fore each ticked alternative please specify what the hazard was and how it affected the patients on-scene.* |

# EMS response data

| | |
|---|---|
| 20 | The following questions are regarding on-scene initial actions by first medical team to arrive on-scene.<br><br>**Did the first medical team to arrive on-scene:**<br>☐ Assume the role of on-scene medical commander?<br>☐ Begin to make an assessment of scene safety?<br>☐ Communicate a situation report to EMS coordinating centre?<br>*If yes was this done according to a pre-existing system or mnemonic? (E.g. METHANE).*<br>☐ Request additional resources?<br>*If yes please specify what types of resources were requested.*<br>☐ Initiate any safety related actions?<br>*If yes please describe which.*<br>☐ Delegate responsibility for other tasks on scene?<br>*If yes please describe other tasks.*<br>For each ticked alternative the responder will be asked to provide time for each action (provided as date: year/month/day and time: hh:mm) and state whether the time provided is exact or estimated.<br><br>**What kind of medical personnel assumed the role of on-scene medical commander?** |
| 21 | The following questions are regarding system-level medical coordination.<br><br>**What time was summoning of additional medical staff to participate in the medical response initiated? (provided as date: year/month/day and time: hh:mm)**<br><br>**Were additional medical staff who responded to the major incident summoned by:**<br>☐ First medical team to arrive on-scene?<br>☐ On-scene medical commander?<br>☐ EMS coordinating centre?<br>☐ Other means? Please specify.<br><br>**Were medical pre-hospital resources used in the major incident response coordinated by:**<br>☐ First medical team to arrive on-scene?<br>☐ On-scene medical commander?<br>☐ EMS coordinating centre?<br>☐ Other means? Please specify.<br><br>**Who was responsible for briefing medical staff of the situation during the pre-hospital major incident medical response?**<br>☐ First medical team to arrive on-scene?<br>☐ On-scene medical commander?<br>☐ EMS coordinating centre?<br>☐ Other. Please specify. |
| 22 | The following questions are regarding medical communication<br><br>**Was communication achieved between medical personnel at the incident:**<br>○ Yes<br>○ No<br>○ Unknown<br><br>*If yes was this communication managed by:*<br>☐ First medical team to arrive on-scene?<br>☐ On-scene medical commander?<br>☐ EMS coordinating centre?<br>☐ Written reports?<br>☐ Other means? Please, specify.<br><br>*If yes, where possible please provide time for initiating the action (provided as date: year/month/day and time: hh:mm).*<br>*If no: why was communication not achieved?*<br><br>**Was communication achieved between the different task forces involved (police, fire fighters, health, political leaders etc)?**<br>○ Yes<br>○ No, between none of the task forces<br>○ Unknown<br><br>*If yes: was communication achieved between all of the task forces or only between some of the task forces?*<br>Follow up questions will be provided to specify between whom it was or was not achieved, and between whom it should have been achieved.<br><br>**Was communication achieved between the scene and the EMS coordinating centre:**<br>○ Yes<br>○ No<br>○ Unknown<br><br>If yes was communication managed by:<br>☐ First medical team to arrive on-scene?<br>☐ On-scene medical commander?<br>☐ EMS coordinating centre?<br>☐ Written reports?<br>☐ Other means? Please, specify.<br>*If yes, where possible please provide time for initiating the action (provided as date: year/month/day and time: hh:mm).*<br>*If no: why was communication not achived?* |

| | |
|---|---|
| | **Was communication achieved between the scene and receiving hospital/s:**<br>○ Yes<br>○ No<br>○ Unknown<br><br>If yes was communication managed by:<br>☐ First medical team to arrive on-scene?<br>☐ On-scene medical commander?<br>☐ EMS coordinating centre?<br>☐ Written reports?<br>☐ Other means? Please, specify.<br>*If yes, where possible please provide time for initiating the action (provided as date: year/month/day and time: hh:mm).*<br>*If no: why was communication not achived?*<br><br>**Was communication achieved between medical response personnel and the general public?**<br>○ Yes<br>○ No<br>○ Unknown<br><br>*If yes was this communication managed by:*<br>☐ First medical team to arrive on-scene?<br>☐ On-scene medical commander?<br>☐ EMS coordinating centre?<br>☐ Written reports?<br>☐ Other means? Please, specify.<br>*If yes, where possible please provide time for initiating the action (date and hh:mm).*<br>*If no: why was communication not achived?* |
| 23 | **Describe the structure of the medical incident command during the major incident (free text).** |
| 24 | The following questions are regarding modes of communication<br><br>**Which mode/s of communication were used during the major incident response? Please tick for all options that apply.**<br>☐ Radio, VHS<br>☐ Radio, tetra<br>☐ Other type of radio<br>☐ Mobile phone<br>☐ Land line telephone<br>☐ Communication to the public (such as television, social media)? Please specify mode of communication<br>☐ Other means of communication. Please specify<br>*For each ticked alternative please state if there were any failures to that mode of communication, specify what the failure was and how it affected the medical response.* |
| 25 | **Are the same communication systems mentioned above in use on a daily basis?**<br>☐ VHF radio<br>☐ Tetra radio<br>☐ Other type of radio<br>☐ Mobile phone<br>☐ Land line telephone<br>☐ Communication to the public (such as television, social media)? Please specify mode of communication<br>☐ Other means of communication. Please specify |
| 26 | **Please provide timings for the following (provided as date: year/month/day. Time: hh:mm):**<br>☐ Incident time<br>☐ Emergency Medical Service (EMS) notification<br>☐ First EMS arrival<br>☐ Major incident declared<br>☐ Medical command established<br>☐ Time of first meeting between police /fire / medical command<br>☐ 1st patient evacuated by EMS (time of leaving incident scene)<br>☐ Last patient evacuated by EMS (time of leaving incident scene)<br>☐ 1st patient arriving in hospital<br>☐ Last patient arriving in hospital<br>*Please state if the timings are estimated or exact.* |
| 27 | **Please describe any delays in the timings mentioned in question 26 (free-text).** |
| 28 | The following questions are regarding on-scene resources<br><br>**What was the number of persons in each category who were present at scene during the EMS response to the incident?**<br>☐ Lay person with no field care education<br>☐ Basic Life Support by non-EMS professional<br>☐ Basic Life Support by EMS professionals, non-physician<br>☐ Advanced Life Support by EMS professional, non-physician<br>☐ Advanced Life Support on-scene by physician<br>☐ Other personnel. Please specify.<br>☐ Unknown<br>*For each ticked option please state whether the number is estimated or exact.* |

| | |
|---|---|
| 29 | **What was the number of units in each transport category that responded to the major incident? Returning units are to be counted only once. Please tick for all options that apply.**<br>☐ EMS: vehicle.<br>☐ EMS: helicopter<br>☐ EMS: boat<br>☐ EMS: other. Please specify type<br>☐ Civilian: vehicle<br>☐ Civilian: helicopter<br>☐ Civilian: boat<br>☐ Civilian: other. Please specify type<br>☐ Other Emergency services: vehicle<br>☐ Other emergency services: helicopter<br>☐ Other emergency services: boat<br>☐ Other emergency services: other means of transport.<br>*If possible, please provide time of arrival for the first vehicle in each category.* |
| 30 | **What kind of equipment was available on-scene enabling EMS to do their job? Please tick for all options that apply:**<br>☐ Equipment to provide care for patients exposed to hazardous materials. Please specify<br>☐ Search and rescue equipment. Please specify<br>☐ Alpine/mountain rescue equipment<br>☐ Coast guard equipment<br>☐ Support vehicles. Please specify<br>☐ Other type of equipment. Please specify<br>*If possible please indicate the time when equipment was ready for use at the scene*<br>*(provided as date: year/month/day and time: hh:mm).* |
| 31 | The following questions are regarding hospitals receiving patients<br><br>**How many hospitals received patients during the major incident?** |
| 32 | For each of the hospitals mentioned in question 31:<br>**What was the distance from incident scene? Distance measured as kilometers in air line.**<br><br>**Type of hospital**<br>☐ Regional hospital with trauma responsibility<br>☐ Regional hospital without trauma responsibility<br>☐ Local hospital<br>☐ Other types of hospitals. Please specify<br>☐ Unknown<br><br>**Number of patients conveyed to hospital:**<br>☐ By EMS<br>☐ By non-EMS<br>☐ In the first hour after the incident (<1 hour)<br>☐ Between 1 and 2 hours after the incident (≥1 hour <2hours)<br>☐ Between 2 and 3 hours after the incident (≥2 hours <3 hours)<br>☐ Between 3 and 4 hours after the incident (≥3 hours < 4 hours)<br>☐ After 4 hours or more following the incident (≥4 hours)<br><br>**Does a pre-existing patient distribution plan exist?**<br>○ Yes<br>○ No<br>○ Unknown<br><br>If yes, please explain any pre-existing patient distribution plan/s and give any comments on decision making, delays etc. |

# Patient characteristics

| | |
|---|---|
| 33 | **What was the estimated number of population at risk from the major incident? (e.g. number of passengers on a train / ship)**<br>Please explain how the above number of population at risk was reached? |
| 34 | **Gender**<br>☐ Males<br>☐ Females<br>☐ Unidentified/missing victims at the time of writing this report<br>*For each category please provide the number and if the numbers are estimated or exact.* |
| 35 | **Were there children requiring the attention of EMS?**<br>○ Yes<br>○ No<br>○ Unknown<br>*If yes number of:*<br>☐ Neonates (0-30 days)<br>☐ Infants (1 month-2 years)<br>☐ Young child (2-6 years)<br>☐ Adolescent (12-18 years)<br>*For each category please state if the number is estimated or exact.* |
| 36 | **What was the number of deaths on-scene before any medical care was provided?** |

| | |
|---|---|
| 37 | **What was the number of deaths after initial treatment, but before transport to hospital was started?** |
| 38 | **What was the number of deaths upon arrival at hospital, but for whom pre-hospital care and transport had been initiated?** |
| 39 | **If available: what was the 30-day mortality of those admitted to hospital?**<br>Please state whether figures are estimated or exact, and if data collection of 30-day mortality of those admitted to hospital is considered complete. |
| 40 | **Was a pre-hospital on- scene triage system used during the major incident response?**<br>○ Yes<br>○ No<br>○ Unknown<br><br>*If yes:*<br>**Who performed the on-scene pre-hospital triage?**<br>☐ Physician<br>☐ EMS personnel<br>☐ Other. Please specify.<br><br>**Which triage system was used?** |
| 41 | **Number of patients in each category upon first assessment on scene**<br>☐ Red = immediate<br>☐ Yellow = urgent<br>☐ Green = minor/delayed<br>☐ Black = deceased<br>☐ Other categories? Please specify.<br>*For each category please specify if the numbers are estimated or exact and please provide the data source from which these numbers originate.* |
| 42 | **Were any patients attended by EMS or medical staff at a primary health care facility and not admitted to hospital?**<br>○ Yes<br>○ No<br>○ Unknown<br>*If yes:*<br>*How many patients sustained minor injuries? Is the number given estimated or exact and from which data source do these numbers originate?* |
| 43 | **Was there any over-or undertriage?**<br>○ *Yes*<br>○ No<br>○ Unknown<br>*If yes: what was the % of overtriage, what was the % of undertriage. Please state any definition for triage precision calculations as well as the data source.* |
| 44 | **What was the total number of patients seeking care at a hospital?**<br><br>**What was the total number of patients admitted to hospital?**<br><br>**How many of the admitted patients were discharged within 24 hours?**<br>*Please state whether figures are estimated or exact and provide the data source (e.g.: hospital records).* |
| 45 | **Did any patients sustain the following types of injury?**<br>☐ Blunt trauma<br>☐ Penetrating trauma<br>☐ Burns<br>☐ Drowning<br>☐ Asphyxiation<br>☐ Hypothermia<br>☐ Intoxication/poisoning<br>☐ Infectious disease<br>☐ Acute psychiatric symptoms<br>☐ Nuclear or radiological injury<br>☐ Biological injury<br>☐ Chemical injury<br>☐ Other types of injury<br>*If possible: for each ticked alternative please specify the number, if the number is estimated or exact and please state the data source for which the numbers derive.* |
| 46 | **Were any patients admitted to critical care area?**<br>○ Yes<br>○ No<br>○ Unknown<br>*If yes:*<br>*Please state the number of patients admitted to critical care area, if the number is estimated or exact and state the data source.*<br>*Please explain how you define critical care.* |

# Key lessons

| | |
|---|---|
| 47 | **During the pre-hospital emergency medical response to this major incident, were there any particular problems that may be improved in future major incidents?**<br>○ Yes<br>○ No<br>*If yes: In what area/s did the problem/s occur?*<br>☐ Issues related to pre-incident situation in the country/region<br>☐ Issues related to EMS situation before the major incident<br>☐ Nature of the incident itself<br>☐ The EMS response<br>☐ Characteristics of the patients<br>☐ Other – please specify |
| 48 | **During the pre-hospital emergency medical response to this major incident, were there any particular successes that may enhance the response to future major incidents?**<br>○ Yes<br>○ No<br>*If yes: In what area/s did the problem/s occur?*<br>☐ Issues related to pre-incident situation in the country/region<br>☐ Issues related to EMS situation before the major incident<br>☐ Nature of the incident itself<br>☐ The EMS response<br>☐ Characteristics of the patients<br>☐ Other – please specify |
